# Statement of Retraction: Gao P, Yang JL, Wang H, Wu XD, Jiao SC. “A two-dimensional model for studying tumor angiogenesis inhibitors”

**DOI:** 10.3109/07357907.2014.969627

**Published:** 2014-10-09

The Editor and Publisher regret to announce the following article published in 2013 has been retracted from publication in *Cancer Investigation:*

Gao P, Yang JL, Wang H, Wu XD, Jiao SC. A two-dimensional model for studying tumor angiogenesis inhibitors. Cancer Invest. 2013 Jun;31(5):346–358.

(doi: 10.3109/07357907.2013.789902). Epub 2013 May 3.

This article has been found to reproduce text and figures to a high degree of similarity, without appropriate attribution or acknowledgement by the authors, from the following original article:

Harrington HA, Maier M, Naidoo L, Whitaker N, Kevrekidis PG. A hybrid model for tumor-induced angiogenesis in the cornea in the presence of inhibitors. Math. Comput. Model. 2007 Aug;46(3–4):513–524. (doi:10.1016/j.mcm.2006.11.034)

The authors have been fully cooperative with our investigations and agree with the Editor and Publisher on this course of action to correct the redundancy in the literature record.

The journal's policy in this respect is clear: *Cancer Investigation* considers all manuscripts on the strict condition that they have been submitted only to *Cancer Investigation,* that they have not been published already, nor are they under consideration for publication or in press elsewhere.

*Cancer Investigation* published this article in good faith, and on the basis of signed statements made by the corresponding author regarding the originality of their work. The article is withdrawn from all print and electronic editions.

Gary H. Lyman (Editor-in-Chief)

Anna Treadway (Publishing Director Journals, Informa Healthcare)

